# Two new species of *Phrynopus* (Anura, Strabomantidae) from high elevations in the Yanachaga-Chemillén National park in Peru (Departamento de Pasco)

**DOI:** 10.3897/zookeys.235.3885

**Published:** 2012-10-31

**Authors:** Edgar Lehr, Jiří Moravec, Juan Carlos Cusi

**Affiliations:** 1 Department of Biology, Illinois Wesleyan University, Bloomington, IL 61701, USA; 2 Department of Zoology, National Museum, 19300 Praha 9, Czech Republic; 3 Departamento de Herpetología, Museo de Historia Natural, Universidad Nacional Mayor de San Marcos. Av. Arenales 1256, Lince, Lima 14, Perú

**Keywords:** Andes, Anura, new species, parental care, Peru, *Phrynopus*, Strabomantidae, Andes, Anura, cuidado parental, especie nueva, Perú, *Phrynopus*, Strabomantidae

## Abstract

We describe two new species of *Phrynopus* from a cloud forest of the Cordillera Yanachaga, Yanachaga-Chemillén National Park in Peru and describe and document the first clutch and case of parental care for a species of *Phrynopus*. One of the new species of *Phrynopus* is described based on two females (SVL 19.1–21.0 mm) which were found in leaf litter and moss layer in a cloud forest at 2900 m elevation. This new species is most similar to *Phrynopus bracki*. The second new species of *Phrynopus* was found in the transitional formation between cloud forest and wet puna at 3000 m elevation. Its description is based on a single female (SVL 20.7 mm) that was observed guarding nine eggs under moss. This new species is most similar to *Phrynopus nicoleae*. The eggs had a diameter of 5.7–5.8 mm (n = 3) and froglets when hatched a SVL of 6.2–6.5 mm (n = 3). Sympatric anurans include *Gastrotheca* sp., *Pristimantis aniptopalmatus*, *Pristimantis bromeliaceus*, *Pristimantis* sp., and *Rhinella yanachaga*.

## Abstract (es)

Se describen dos nuevas especies de *Phrynopus* procedentes de un bosque nublado de la Cordillera Yanachaga, Parque Nacional Yanachaga-Chemillén, Perú, y se describe y documenta el primer caso de nidada y cuidado parental para una especie de *Phrynopus*. Una de las nuevas especies de *Phrynopus* es descrita basándose en dos hembras (Longitud Hocico-Cloaca 19.1–21.0 mm). Los especímenes fueron encontrados en la hojarasca y capa de musgo en un bosque nublado a 2900 m de altitud. Esta especie es muy similar a *Phrynopus bracki*. La segunda nueva especie de *Phrynopus* fue encontrada en una formación transicional entre el bosque nublado y la puna humeda a 3000 m de altitud. Su descripción se basa en una sola hembra (Longitud Hocico-Cloaca 20.7 mm) que fue observada protegiendo nueve huevos bajo musgo. Esta especie es muy similar a *Phrynopus nicoleae*. Los huevos tenían un diámetro de 5.7–5.8 mm (n = 3) y las ranitas cuando eclosionaron tenían una longitud hocico-cloaca de 6.2–6.5 mm (n = 3). Los anuros simpátricos incluyen *Gastrotheca* sp., *Pristimantis aniptopalmatus*, *Pristimantis bromeliaceus*, *Pristimantis* sp.y *Rhinella yanachaga*.

## Introduction

**Figure 1.**
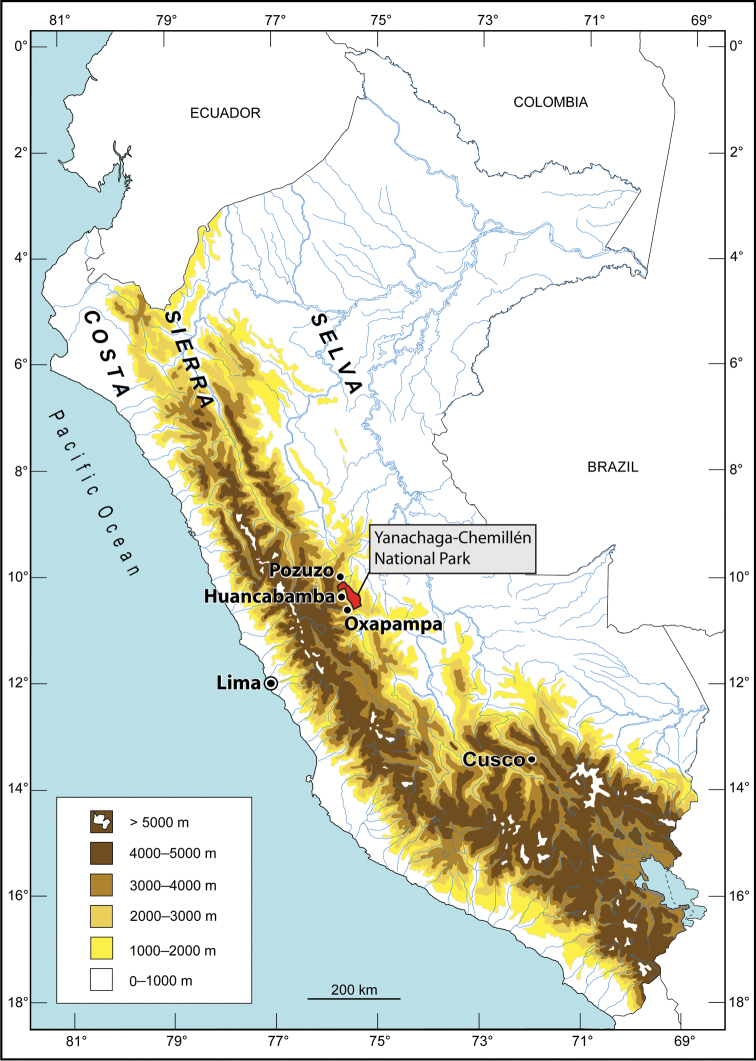
Map of Peru with the Yanachaga-Chemillén National Park in red. Map by E. Lehr.

**Figure 2.**
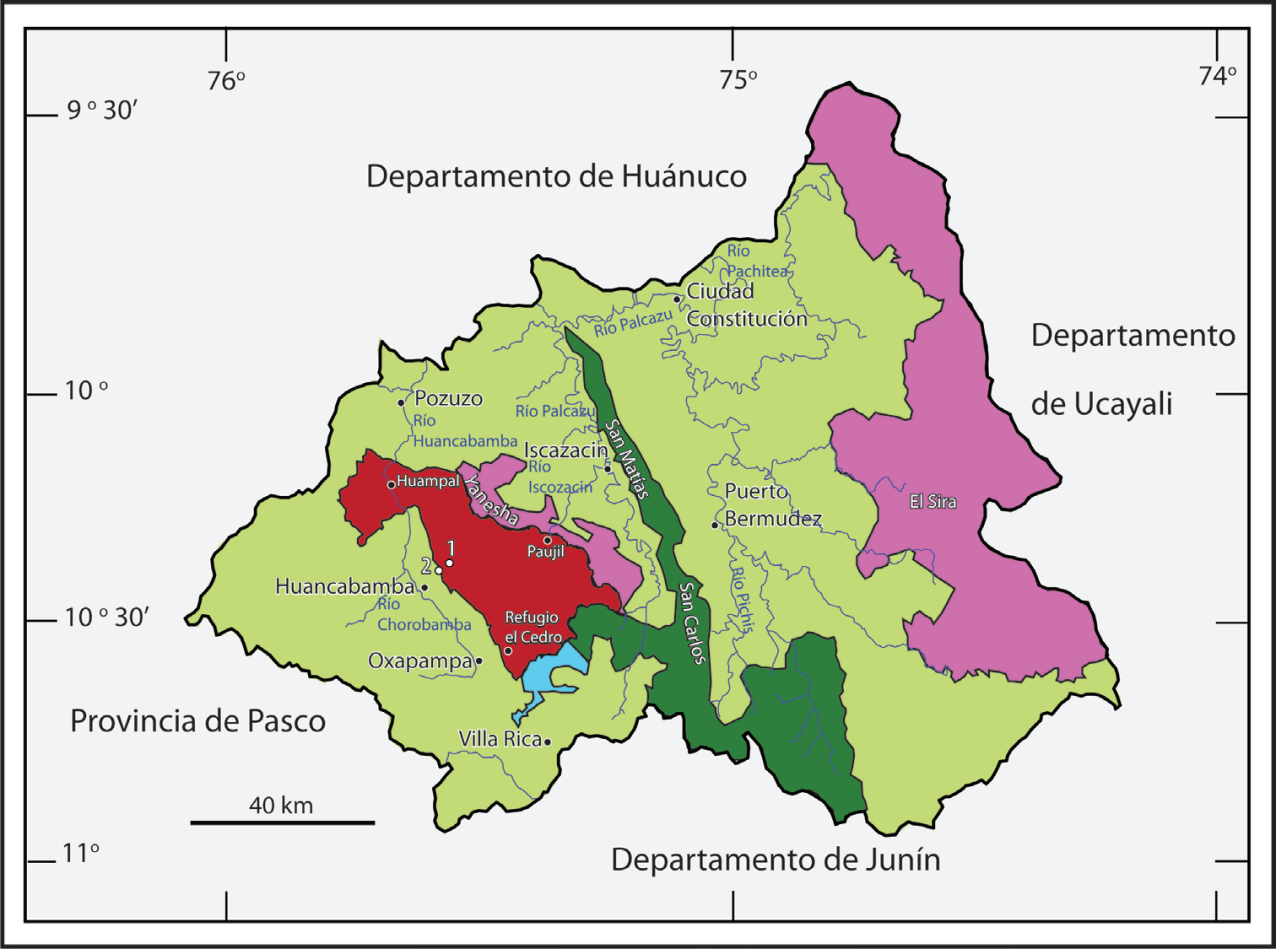
The Yanachaga-Chemillén National Park (in red) with three field stations: Refugio el Cedro at 2400 m, Huampal at 1000 m, and Paujil at 460 m. Other protected areas in the Central Selva region: in pink, Communal Reserves (Yanesha, El Sira); in dark, green Protected Forest (San Matías-San Carlos); and in blue, Municipal Conservation Area (Bosque de Sho’llet). Unprotected Provincial Area is shown in pale green. Collecting sites: 1, Quebrada Yanachaga (2900–3000 m); 2, Huancabamba park entrance (2290–2350 m). Designed by E. Lehr using a template from The Nature Conservancy (2007).

**Figure 3.**
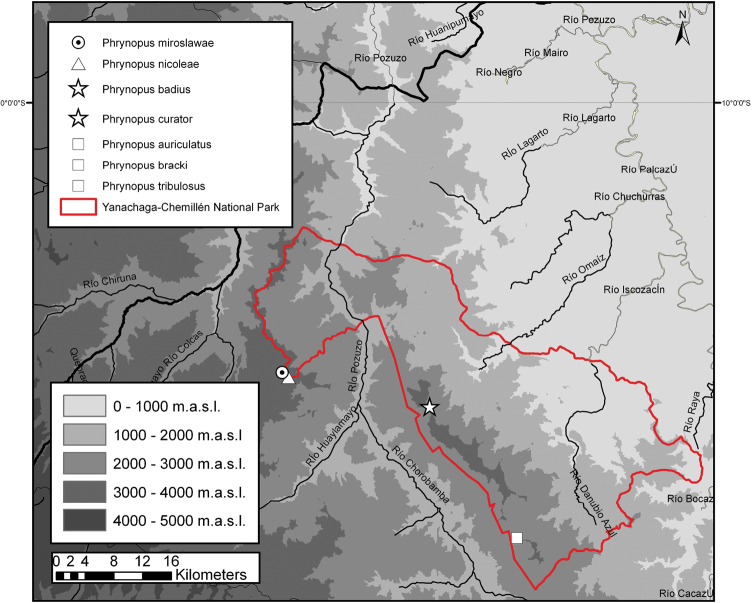
Distribution (based on type localities) of the seven species of *Phrynopus* known from the Yanachaga-Chemillén National Park. Map by J. C. Cusi.

The Yanachaga-Chemillén National Park (YCNP in the following) is located in the eastern Andes of the poorly explored Pasco department (Figs 1, 2). It was established in 1986 with the aim to protect a unique biodiversity area of the Andean montane cloud forest. Owing a difficult access to the area, the flora and fauna of the YCNP remained mostly unexplored until recently (Yallico and Rose 1998). Knowledge regarding the floristic composition of YCNP is steadily increasing especially due to the efforts of the botanical inventory and educational programs conducted by the Missouri Botanical Garden (e.g., Jasmín and Churchill 2008; La-Torre et al. 2003). More than 2000 plants have been identified within the territory of the YCNP (Brack et al. 2010). Among the vertebrate fauna, 527 species of birds and 49 species of mammals have been recorded inside the YCNP (Brack et al. 2010), but exploration of the herpetofauna has mostly been neglected. The Instituto Nacional de Recursos Naturales (1995) reported 16 species of reptiles and 2 species of frogs from the YCNP, but did not list these species. The few herpetological surveys that were conducted inside the YCNP in the 1980s by Hedges, early 1990s by Icochea, or in 2008 by Chaparro and by Boano and colleagues had a focus on amphibians, and lasted between a few days to a few weeks. A total of six new species of frogs (*Phrynopus*, *Pristimantis*, Strabomantidae) from the montane forest of YCNP were described by Hedges (1990) and Duellman and Hedges (2005, 2007, 2008). The fieldwork by Chaparro et al. (2008) led to the description of two new species of *Phrynopus* from the puna of the YCNP (Fig. 3). Duellman and Chaparro (2008) described two new species of *Pristimantis* from the puna and cloud forest of the YCNP, and Boano et al. (2008) described a new species of *Pristimantis* from the field station Refugio El Cedro. Lehr reviewed the amphibian specimens collected by Icochea deposited at the Natural History Museum in Lima (MUSM). This revision led to the description of three new species of anurans (*Gastrotheca carinaceps* Duellman, Trueb and Lehr, 2006; *Melanophryne barbatula* Lehr and Trueb, 2007; and *Rhinella yanachaga* Lehr, Pramuk, Hedges and Córdova, 2007). In summary, 16 new species of amphibians from the YCNP have been described since 1990 (see table 1). However, according to Brack et al. (2010) 71 species of amphibians and 44 species of reptiles were registered in the YCNP during an inventory in 2008. Nevertheless, no species lists of amphibians and reptiles are available nor any information regarding the deposition of voucher specimens. In an attempt to increase the knowledge of the local herpetofauna further, we explored areas within the YCNP that had not been surveyed previously, such as the Quebrada Yanachaga (Figs 2, 3). This led to the discovery of new species of anurans, among them two new species of *Phrynopus* that are described herein.

**Table 1.** Amphibian species described from the YCNP since 1990.

**Family**	**Species**	**Publication**	**Ecoregion**
Bufonidae	*Rhinella yanachaga*	Lehr et al. (2007)	Montane forest (2600 m)
Hemiphractidae	*Gastrotheca carinaceps*	Duellman et al. (2006)	unknown
Microhylidae	*Melanophryne barbatula*	Lehr and Trueb (2007)	Montane forest (2500 m)
Strabomantidae	*Phrynopus auriculatus*	Duellman and Hedges (2008)	Montane forest (2600 m)
Strabomantidae	*Phrynopus badius*	This paper	Montane forest (2900 m)
Strabomantidae	*Phrynopus bracki*	Hedges (1990)	Montane forest (2600 m)
Strabomantidae	*Phrynopus curator*	This paper	Montane forest (3000 m)
Strabomantidae	*Phrynopus miroslawae*	Chaparro et al. (2008)	Puna (3363 m)
Strabomantidae	*Phrynopus nicoleae*	Chaparro et al. (2008)	Puna (3589 m)
Strabomantidae	*Phrynopus tribulosus*	Duellman and Hedges (2008)	Montane forest (2600)
Strabomantidae	*Pristimantis albertus*	Duellman and Hedges (2007)	Montane forest (1970 m)
Strabomantidae	*Phrynopus aniptopalmatus*	Duellman and Hedges (2005)	Montane forest (2300–2600 m)
Strabomantidae	*Phrynopus bipunctatus*	Duellman and Hedges (2005)	Montane forest (2060–2120 m)
Strabomantidae	*Phrynopus leucorrhinus*	Boano et al. (2008)	Montane forest (2500 m)
Strabomantidae	*Phrynopus lucasi*	Duellman and Chaparro (2008)	Montane forest (2790 m)
Strabomantidae	*Phrynopus rhabdocnemus*	Duellman and Hedges (2005)	Montane forest (2600 m)
Strabomantidae	*Phrynopus stictogaster*	Duellman and Hedges (2005)	Montane forest (2600 m)
Strabomantidae	*Phrynopus spectabilis*	Duellman and Chaparro (2008)	Puna (3300 m)

## Methods

### Study Area

The YCNP (Figs 1–3) covers 1220 km² between 460 and 3643 m elevation and is located at 10°33’37”-17’37”S and 75°30’21”-20’39”W in eastern central Peru (Yallico and Rose 1998). It is contiguous with three other protected areas, the San Matías-San Carlos Protected Forest (1458 km²), the Yanesha Communal Reserve (347 km²), and the Municipal Conservation “Area Bosque de Sho’llet” (14 km²) covering together 3039 km² (The Nature Conservancy 2007, Brack et al. 2010). Located geographically inside the national park is the Cordillera Yanachaga, a mountain range east of, and isolated from, the eastern chain of the Andes. Environmental gradients within the YCNP span the ecoregions of puna (3000-3643 m), montane forests (600-3000 m), often separated by Inter Andean valleys, and humid tropical forests (below 600 m).

The Quebrada Yanachaga lies at the southwestern edge of the YCNP (ca. 10-15 km NE from Huancabamba, Fig. 2) at an approximate elevation between 2000 and 3000 m. It is covered by a dense primary montane cloud forest, which changes into more open formation called “pajonal” at the elevation of ca. 2900 m. Quebrada Yanachaga can be reached from Huancabamba taking the road to Prosoya (former Hacienda, see Lehr and von May 2004) and then walking towards the entrance of the park following a narrow trail. The field survey was conducted during January 16-23, 2012.

### Morphological characters

The format for the description follows Lynch and Duellman (1997), and diagnostic characters of Duellman and Lehr (2009). Specimens were preserved in 96% ethanol and stored in 70% ethanol. Specimens were dissected to determine sex and maturity. The senior author measured the following variables to the nearest 0.1 mm with digital calipers under a microscope: snout–vent length (SVL), tibia length (TL), foot length (FL, distance from proximal margin of inner metatarsal tubercle to tip of Toe IV), head length (HL, from angle of jaw to tip of snout), head width (HW, at level of angle of jaw), eye diameter (ED), interorbital distance (IOD), upper eyelid width (EW), internarial distance (IND), and eye–nostril distance (E-N, straight line distance between anterior corner of orbit and posterior margin of external nares). Fingers and toes are numbered preaxially to postaxially from I–IV and I–V respectively. We determined comparative lengths of Toes III and V by adpressing both toes against Toe IV; lengths of Fingers I and II were determined by adpressing the fingers against each other. To reduce reflections, preserved holotypes were photographed submersed in ethanol including ventral surfaces of hands and feet. Photographs taken in the field by E. Lehr and J. Moravec were used for descriptions of color in life.

Specimens were deposited in the herpetological collections of the Museo de Historia Natural, Universidad Nacional Mayor de San Marcos (MUSM) in Lima, Peru, and the Field Museum (FMNH) in Chicago. For specimens examined, see Appendix I.

## Results

### Phrynopus badius sp. n.

urn:lsid:zoobank.org:act:DB17180B-BD8F-40AB-80B6-B472E9D29F81

http://species-id.net/wiki/Phrynopus_badius

#### Holotype.

**Figure 4.**
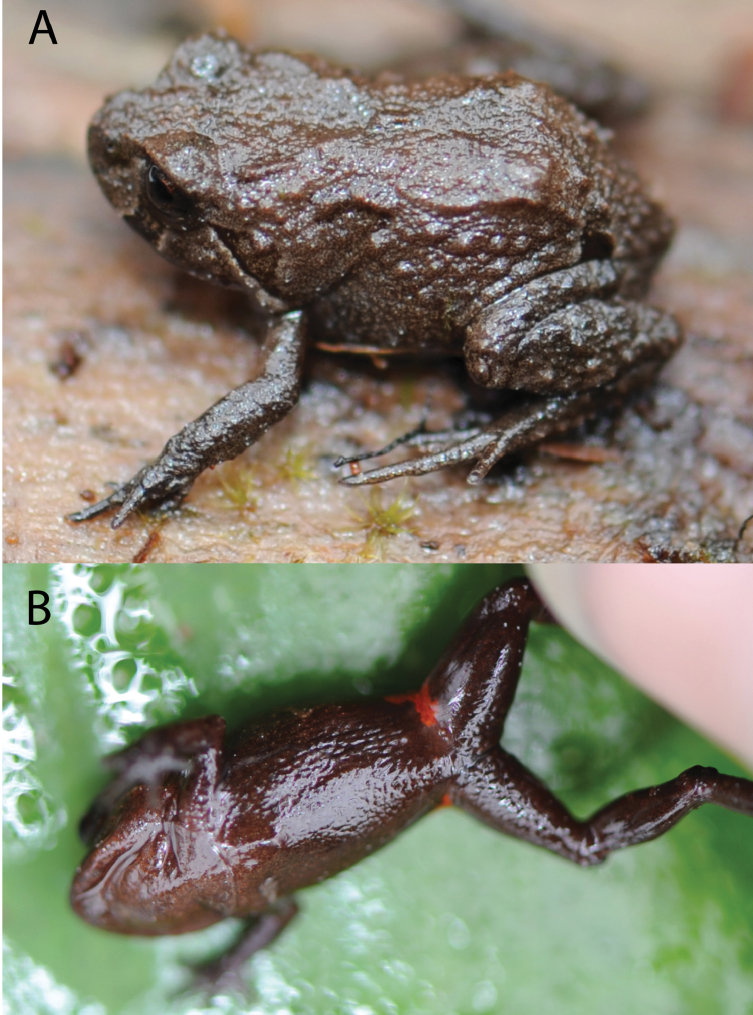
Holotype of *Phrynopus badius* in life (MUSM 31099, female, SVL 19.1 mm) in lateral (**A**), and ventral views (**B**). Photos by E. Lehr.

**Figure 5.**
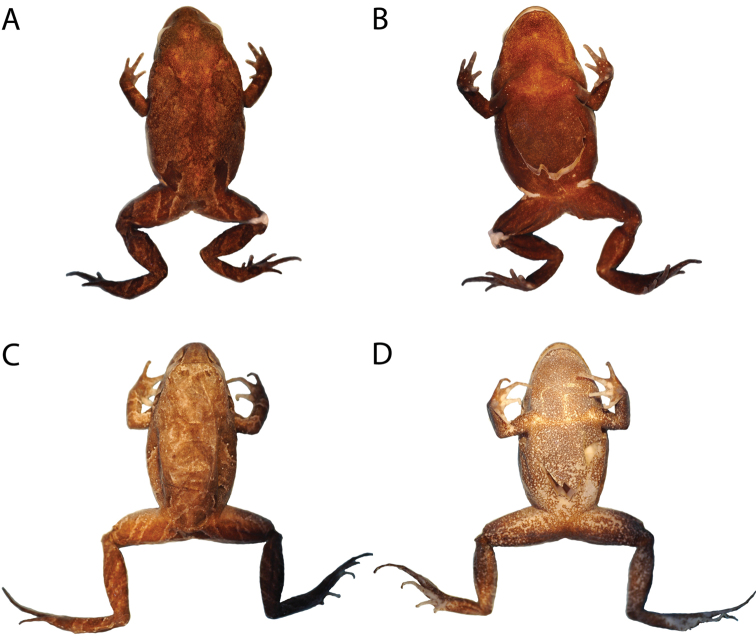
Holotype of *Phrynopus badius* in preservative in dorsal (**A**), and ventral (**B**) views, and holotype of *Phrynopus curator* in dorsal (**C**), and ventral (**D**) views. Photos by E. Lehr.

**Figure 6.**
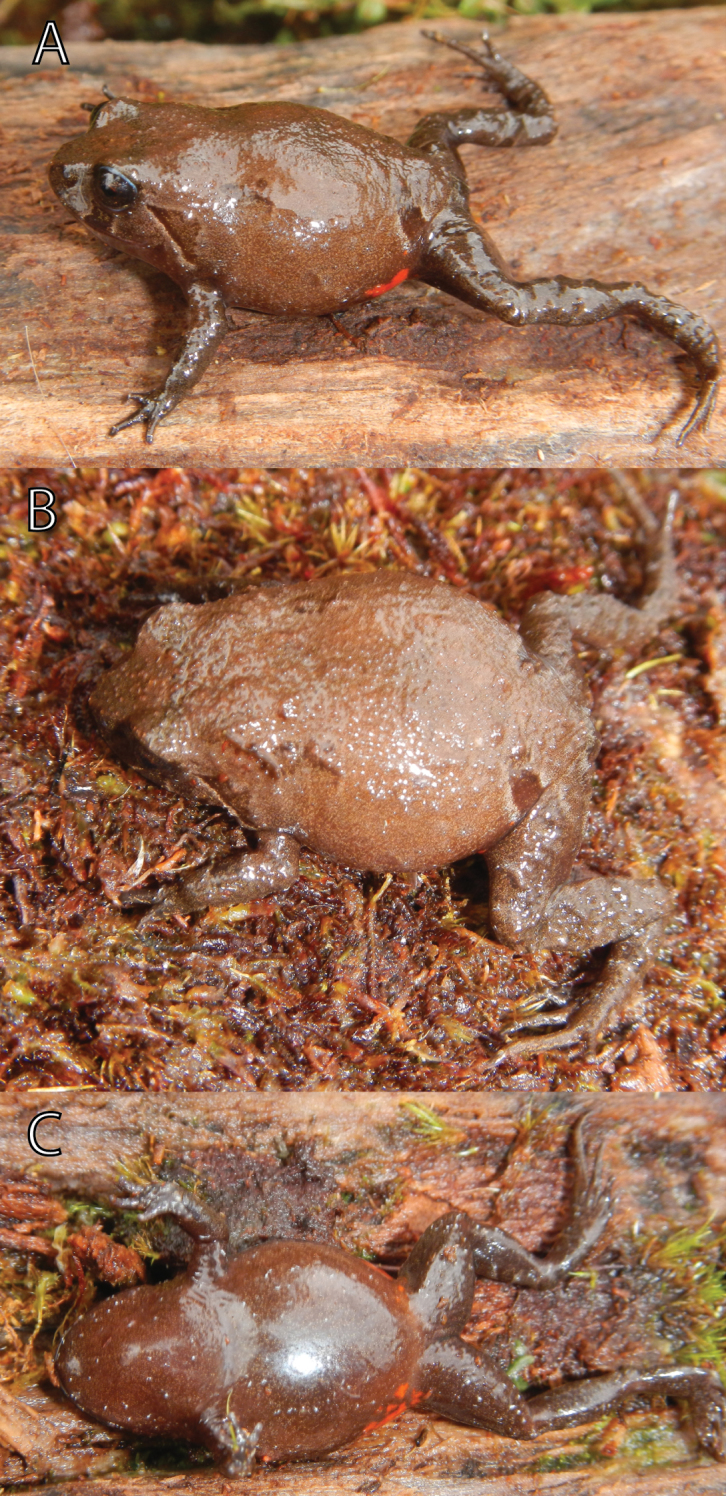
Paratype of *Phrynopus badius* in life (FMNH 282818, female, SVL 21.0 mm) in lateral (**A**), dorsal (**B**), and ventral (**C**) views. Photos by E. Lehr.

**Figure 7.**
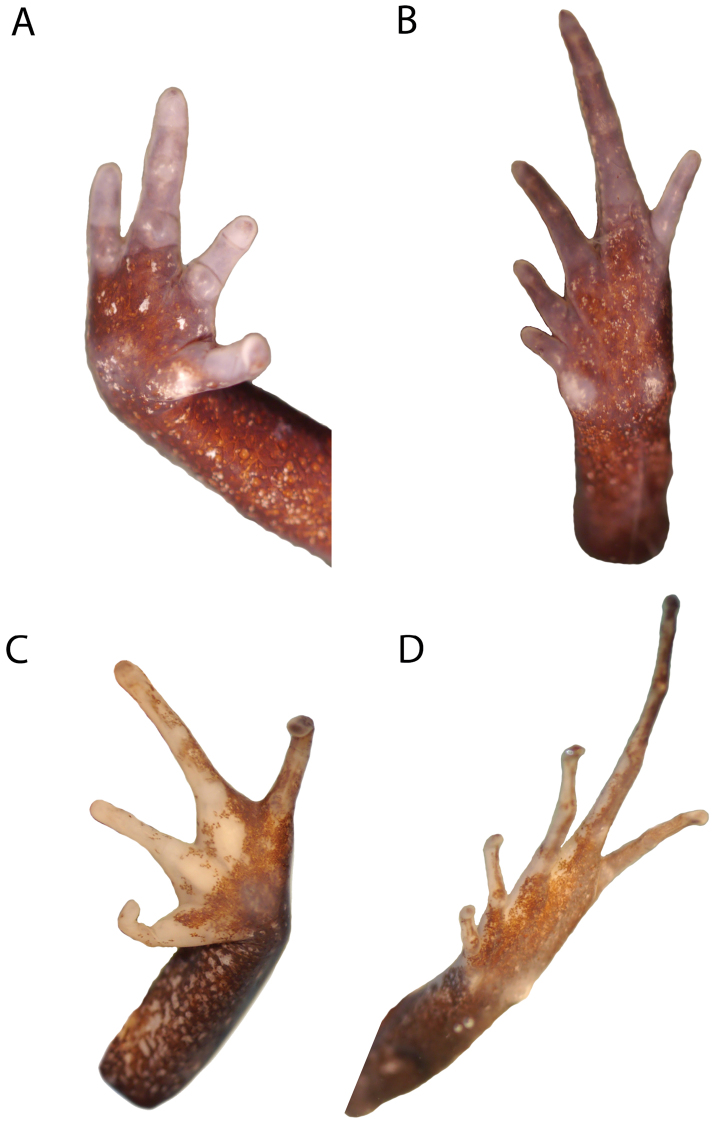
Photos of ventral surfaces of hand (**A**) and foot (**B**) of *Phrynopus badius* (MUSM 31099), and ventral surfaces of hand (**C**) and foot (**D**) of *Phrynopus curator* (MUSM 31106). Photos by E. Lehr.

(Figs 4–7) MUSM 31099, an adult gravid female from Quebrada Yanachaga (ca. 10°22.772'S, 75°27.717'W), 2900 m elevation, Yanachaga-Chemillén National Park (Sector San Daniel), Distrito de Huancabamba, Provincia de Oxapampa, Departamento de Pasco, Peru, collected on 19 January 2012 by Edgar Lehr, Jiri Moravec, and Juan Carlos Cusi.

#### Paratype.

FMNH 282818, an adult, gravid female, collected along with the holotype.

#### Diagnosis.

A species of *Phrynopus* having the following combination of characters: (1) Skin on dorsum shagreen with small scattered tubercles, flanks tuberculate, skin on venter weakly areolate; discoidal fold absent, thoracic fold present; short postocular fold present, elongate tubercles forming discontinous dorsolateral ridges; (2) tympanic membrane and tympanic annulus absent; (3) snout rounded in dorsal and lateral views; (4) upper eyelid without enlarged tubercles; width of upper eyelid narrower than IOD; cranial crests absent; (5) dentigerous processes of vomers absent; (6) condition of vocal slits and nuptial pads unknown (no males found); (7) Finger I shorter than Finger II; tips of digits rounded; (8) fingers without lateral fringes; (9) ulnar and tarsal tubercles absent; (10) heel with minute tubercles; inner tarsal fold absent; (11) inner metatarsal tubercle ovoid, about twice as large as rounded outer metatarsal tubercle; supernumerary plantar tubercles absent; (12) toes without lateral fringes; basal webbing absent; Toe V shorter than Toe III; toe tips rounded (except for slightly pointed Toe IV), about as large as those on fingers; (13) in life, dorsum reddish brown or dark grayish brown, venter dark brown with scattered minute white dots, groin dark brown with bright orange flecks on its lower half and a dark brown inguinal bar on its upper half; (14) SVL in females 19.1–21.0 mm (n = 2).

The assignment of the new species to *Phrynopus* is based on the structure of the digital discs that lack circumferential groves as well as the overall morphological similarity with the other members of the genus. *Phrynopus badius* is readily distinguished from its congeners by its small size, by having discontinuous dorsolateral ridges, and by having the dorsum reddish brown or dark grayish brown and the venter dark brown with scattered minute white dots, groin dark brown with bright orange flecks on its lower half and a dark brown inguinal bar on its upper half.

Furthermore *Phrynopus badius* differs from those species of *Phrynopus* (*auriculatus, montium, peruanus*) that have a tympanum (absent in *Phrynopus badius*), and from those species (*dagmarae, horstpauli, kotosh, miroslawae, nicoleae, vestigiatus*) that have dentigerous processes of vomers (absent in *Phrynopus badius*). *Phrynopus badius* shares with eight other species of *Phrynopus* (*bracki, dagmarae, heimorum, interstinctus, nicoleae, paucari, peruanus, vestigiatus*) an aposematic coloration consisting of red, orange, salmon or flesh coloured blotches in the groin. However, none of these species has the venter dark brown with scattered minute white dots, groin dark brown with bright orange flecks on its lower half, and a dark brown inguinal bar on its upper half.

Six other species of *Phrynopus* have been recorded from the YCNP. Those are *Phrynopus auriculatus* (Duellman and Hedges 2008, at 2600 m), *Phrynopus bracki* (Hedges 1990, at 2600 m), *Phrynopus curator* sp. n. (this paper, 3000 m), *Phrynopus miroslawae* (Chaparro et al. 2008, at 3363 m), *Phrynopus nicoleae* (Chaparro et al. 2008, at 3589 m),and *Phrynopus tribulosus* (Duellman and Hedges 2008, at 2600 m). *Phrynopus miroslawae* and *Phrynopus nicoleae* are from the puna of Santa Bárbara (Chaparro et al. 2008) which is located west of the Río Pozuzo, whereas all others are recorded east of the Río Huancabamba (see Fig. 3). *Phrynopus badius* lacks a tympanum (present in *Phrynopus auriculatus*), does not have Toe I vestigial (vestigial in *Phrynopus bracki*), has groin dark brown with orange flecks on its lower half and a dark inguinal bar on its upper half (groin grayish brown in *Phrynopus curator*), lacks X-shaped dorsal ride and dentigerous processes of vomers (both present in *Phrynopus nicoleae*), lacks dorsolateral folds (prominent in *Phrynopus miroslawae*), and has the dorsum reddish brown or dark grayish brown (green in *Phrynopus tribulosus*). *Phrynopus badius* and *Phrynopus bracki* are similar in being small (SVL 21.0 mm in *Phrynopus badius* vs. 19.8 mm in *Phrynopus bracki*, Duellman and Lehr, 2009) and in having a predominately dark brown coloration. However, both can be distinguished as follows: *Phrynopus badius* lacks a discoidal fold and dentigerous processes of vomers (both present in *Phrynopus bracki*), fingers without lateral fringes (present in *Phrynopus bracki*), ulnar tubercles not coalesced into a ridge (ridge present in *Phrynopus bracki*), Toe V shorter than Toe III (Toe V longer than Toe III in *Phrynopus bracki*), Toe I longer than wide (Toe I as long as wide, vestigial in *Phrynopus bracki*), iris predominately black with fine bronze reticulations and red ringlet around pupil (iris bronze in *Phrynopus bracki*).

#### Description of holotype.

Head narrower than body, slightly wider than long, HW 101.2% of HL; HW 34.0% of SVL; HL 33.5% of SVL; snout short, rounded in dorsal and lateral views (Figs 4, 5), ED larger than E-N distance; nostrils protuberant, directed dorsolaterally; canthus rostralis short, slightly concave in dorsal view, rounded in profile; loreal region slightly concave; lips rounded; upper eyelid without enlarged tubercles; EW narrower than IOD (EW 60.9% of IOD); supratympanic fold short and low, extending from posterior corner of eye to level of jaw articulation, barely distinguishable in preservation; tympanic membrane and tympanic annulus absent; postrictal tubercles indistinguishable from surrounding tuberculation. Choanae small, ovoid, not concealed by palatal shelf of maxilla; dentigerous processes of vomers absent; tongue narrow and long, much longer than wide, not notched posteriorly, posterior half free.

Skin on dorsum shagreen with small scattered tubercles, short postocular fold present, elongate tubercles forming discontinuous dorsolateral ridges; skin on flanks tuberculate; skin on throat smooth, that on chest and belly weakly areolate; discoidal fold absent, thoracic fold present; cloacal sheath short; large tubercles absent in cloacal region. Outer surface of forearm without minute tubercles; outer and inner palmar tubercles low, ovoid, outer about twice the size of inner palmar tubercle; supernumerary tubercles indistinct in preservative; subarticular tubercles low, barely discernible except for prominent, ovoid subarticular tubercles on base of fingers; fingers without lateral fringes; Finger I shorter than Finger II; tips of digits rounded, lacking marginal grooves (Fig. 7A).

Hind limbs short and robust, TL 37.2% of SVL; FL 41.4.9% of SVL; upper surface of hind limbs tuberculate; posterior and ventral surfaces of thighs areolate; heel with minute tubercles; outer surface of tarsus with small tubercles; inner metatarsal tubercle ovoid, about twice as large as rounded outer metatarsal tubercle; supernumerary plantar tubercles absent; subarticular tubercles low, ovoid in dorsal view; toes without lateral fringes; basal webbing absent; toe tips rounded (except for pointed tip of Toe IV), lacking marginal grooves, about as large as those on fingers; relative lengths of toes: 1 < 2 < 3 > 5 < 4; Toe V shorter than Toe III, Toe I less than half the size of Toe II (Fig. 7B).

Measurements (in mm) of holotype:SVL 19.1; TL 7.1; FL 7.9; HL 6.4; HW 6.5; ED 2.1; IOD 2.3; EW 1.4; IND 1.9; E-N 1.5.

#### Coloration of holotype in life.

(Fig. 4): Dorsum dark grayish brown with white dots and reddish brown mottling; narrow, dark brown interorbital bar and a dark brown blotch on each side in the scapular region; dorsal surface of forearms with a dark brown bar; hind legs each with three dark brown diagonal stripes; ill-defined dark brown canthal stripe, supratympanic stripe broad, dark brown and outlined by tan; broad, dark brown bar on upper lip below eye bordered by narrow, tan stripe on each side; flanks coloured as dorsum with broad diagonal stripe at the anterior half; axilla dark brown with a small orange fleck; groin dark brown with orange flecks on its lower half and a dark inguinal bar on its upper half extending onto dorsal surface of thigh; posterior surfaces of thighs and concealed surfaces of shanks dark brown with few white dots; throat reddish brown with white dots; chest, belly, and extremities dark brown with white dots; fingers and toes gray, palmar and plantar surfaces gray with white mottling; iris predominately black with fine bronze reticulations and red ringlet around pupil.

#### Coloration of holotype in preservative.

(Figs 5A, B): As described above but slightly paler and orange coloration being white; iris gray.

#### Variation.

The female paratype (Fig. 6) is larger than the holotype, less tuberculate, and has elongate tubercles forming ill-defined dorsolateral ridges restricted to the anterior half of its body. The overall coloration pattern is similar to the holotype, however the dorsal coloration is reddish brown, the venter has more white dots and the groin has more orange flecks.

Measurements (in mm) of female (FMNH 282818): SVL 21.0; TL 7.5; FL 8.5; HL 7.0; HW 7.6; ED 2.3; IOD 2.4; EW 1.4; IND 1.9; E-N 1.7.

#### Etymology.

The specific name *badius* is the Latin adjective meaning chestnut-brown or reddish-brown and refers to the coloration of the new species.

#### Distribution, ecology, and threat status.

The species is only known from the type locality where it was found at 1.00 pm in the leaf litter and under moss in a primary cloud forest at 2900 m elevation. The axilla region of the left arm of the paratype bears two ectoparasites (mites). Syntopic anurans include *Rhinella yanachaga* (MUSM 31100, FMNH 282819) which is recorded herein for the first time outside its type locality of San Alberto (Lehr et al. 2007). We classify *Phrynopus badius* as “Data Deficient” according to the IUCN red list criteria and categories based on the limited information on its geographic range.

### Phrynopus curator sp. n.

urn:lsid:zoobank.org:act:E64899F5-B179-4A45-8E8A-CD6D8BB977ED

http://species-id.net/wiki/Phrynopus_curator

#### Holotype.

**Figure 8.**
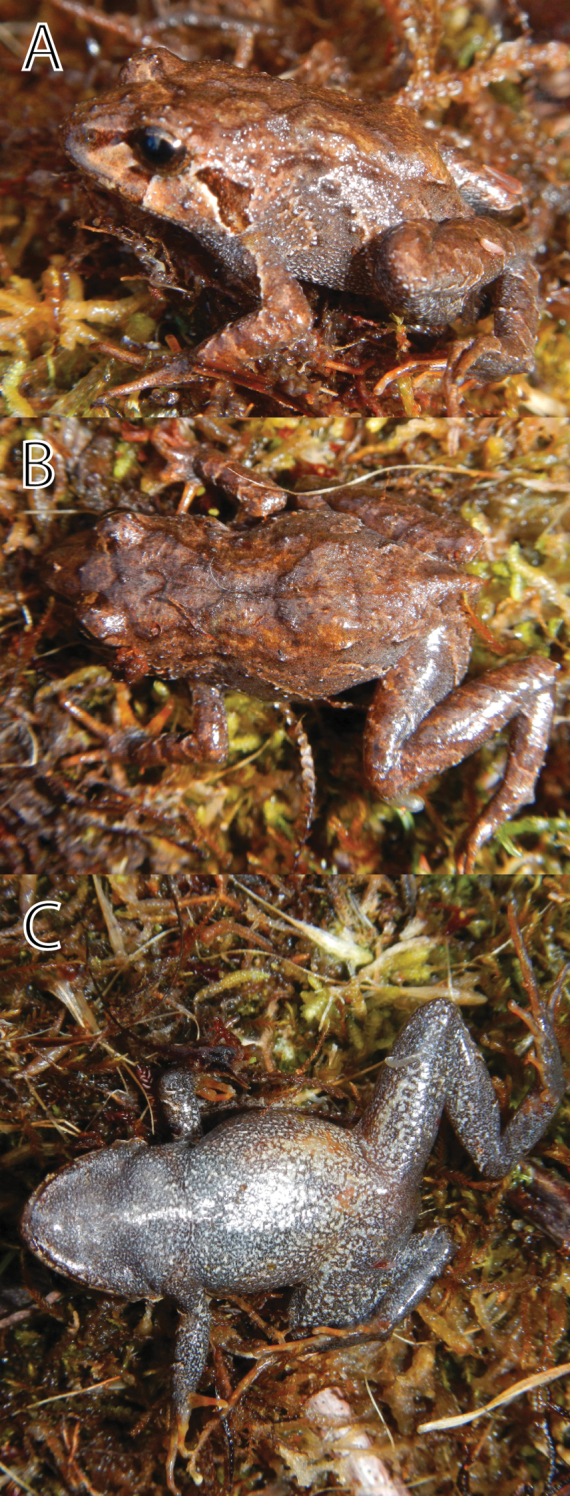
Holotype of *Phrynopus curator* in life (MUSM 31106, female, SVL 20.7 mm) in lateral (**A**), dorsal (**B**), and ventral views (**C**). Photos by E. Lehr.

(Figs 5, 7, 8, 10) MUSM 31106, an adult, gravid female, from Quebrada Yanachaga (10°22.772'S, 75°27.717'W), 3000 m elevation, Yanachaga-Chemillén National Park (Sector San Daniel), Distrito de Huancabamba, Provincia de Oxapampa, Departamento de Pasco, Peru, collected on 20 January 2012 by Edgar Lehr, Jiri Moravec, and Juan Carlos Cusi.

#### Referred specimens.

(Fig. 10C) FMNH 282820-22, three hatchlings, collected as eggs with the holotype.

#### Diagnosis.

A species of *Phrynopus* having the following combination of characters: (1) Skin on dorsum shagreen with small scattered tubercles, prominent ridges, and two prominent middorsal Y-shaped ridges, flanks shagreen with small scattered tubercles, skin on venter weakly areolate; discoidal fold absent, weak thoracic fold present; ridges forming discontinuous dorsolateral fold; (2) tympanic membrane and tympanic annulus absent; (3) snout rounded in dorsal and lateral views; (4) upper eyelid with three enlarged tubercles; width of upper eyelid narrower than IOD; cranial crests absent; (5) dentigerous processes of vomers absent; (6) condition of vocal slits and nuptial pads unknown (no males found); (7) Finger I shorter than Finger II; tips of digits rounded; (8) fingers without lateral fringes; (9) ulnar and tarsal tubercles absent; (10) heel with a distinct conical tubercle; inner tarsal fold absent; (11) outer metatarsal tubercle rounded, about twice as large as ovoid inner metatarsal tubercle; supernumerary plantar tubercles absent; (12) toes without lateral fringes; basal webbing absent; Toe V slightly shorter than Toe III; toe tips rounded, about as large as those on fingers; (13) in life, dorsum reddish brown with dark gray and yellowish-brown mottling, venter gray with pale gray mottling and brownish-cream flecks around posterior half of belly, groin brown and gray mottled; (14) SVL in single female 20.7 mm.

The assignment of the new species to *Phrynopus* is based on the structure of the digital discs that lack circumferential groves as well as the overall morphological similarity with the other members of the genus. *Phrynopus curator* is readily distinguished from its congeners by having the dorsum with prominent middorsal Y-shaped ridges, a conical heel tubercle, absence of dentigerous processes of vomers, and a gray venter with pale gray mottling.

Furthermore *Phrynopus curator* differs from those species of *Phrynopus* (*auriculatus, montium*, and *peruanus*) that have a tympanum (absent in *Phrynopus curator*), and from those species (*dagmarae, horstpauli, kotosh, miroslawae, nicoleae*, and *vestigiatus*) that have dentigerous processes of vomers (absent in *Phrynopus curator*). *Phrynopus curator* lacks an aposematic coloration consisting of red, orange, salmon or flesh colored blotches in the groin (present in *badius, bracki, dagmarae, heimorum, interstinctus, nicoleae, paucari, peruanus*, and *vestigiatus*).

Six other species of *Phrynopus* have been recorded from the YCNP. Those are *Phrynopus auriculatus* (Duellman and Hedges 2008, at 2600 m), *Phrynopus badius* (this paper, 2900 m), *Phrynopus bracki* (Hedges 1990, at 2600 m), *Phrynopus miroslawae* (Chaparro et al. 2008, at 3363 m), *Phrynopus nicoleae* (Chaparro et al. 2008, at 3589 m),and *Phrynopus tribulosus* (Duellman and Hedges 2008, at 2600 m). *Phrynopus miroslawae* and *Phrynopus nicoleae* are from the puna of Santa Bárbara (Chaparro et al. 2008) which is located west of the Río Huancabamba, whereas all others are recorded east of the Río Huancabamba (see Fig. 3). *Phrynopus curator* lacks a tympanum (present in *Phrynopus auriculatus*), has the groin brown and gray mottled (groin dark brown with orange flecks on its lower half and a dark inguinal bar on its upper half in *Phrynopus badius*), does not have Toe I vestigial (vestigial in *Phrynopus bracki*), and its Finger I is shorter than Finger II (Finger I and Finger II of equal length in *Phrynopus nicoleae*). Furthermore *Phrynopus curator* lacks dentigerous processes of vomers (present in *Phrynopus nicoleae*), it has three enlarged tubercles on the upper eyelid (upper eyelid without enlarged tubercles in *Phrynopus nicoleae*), its flanks are reddish brown with dark gray and yellowish-brown mottling and with a broad gray diagonal stripe at the anterior half, bordered on both sides with a narrow tan stripe (flanks tan with abundant bluish-white spots in *Phrynopus nicoleae*), and its groin is brown and gray mottled (tan with abundant bluish-white spots and an orange spot in *Phrynopus nicoleae*). *Phrynopus curator* is smaller than *Phrynopus miroslawae* (SVL 20.7 mm vs. 29.2 mm in *Phrynopus miroslawae*, Chaparro et al. 2008), has dorsum with ridges (dorsum warty in *Phrynopus miroslawae*), and dorsolateral folds discontinuous (continuous), and has the dorsum reddish brown with dark gray and pale brown mottling (green in *Phrynopus tribulosus*).

#### Description of holotype. 

Head narrower than body, slightly longer than wide, HW 98.6% of HL; HW 33.8% of SVL; HL 33.3% of SVL; snout moderately long, rounded in dorsal and lateral views (Figs 5, 8), ED larger than E-N distance; nostrils not protuberant, directed dorsolaterally; canthus rostralis short, straight in dorsal view, rounded in profile; loreal region slightly concave; lips rounded; upper eyelid without enlarged tubercles; EW narrower than IOD (EW 82.6% of IOD); supratympanic fold short and low, extending from posterior corner of eye to level of jaw articulation, barely distinguishable in preservation; tympanic membrane and tympanic annulus absent; minute postrictal tubercles. Choanae small, ovoid, not concealed by palatal shelf of maxilla; dentigerous processes of vomers absent; tongue broad, longer than wide, not notched posteriorly, posterior one third free.

Skin on dorsum shagreen with small scattered tubercles, prominent ridges, and two prominent middorsal Y-shaped ridges connected at their bases; postocular folds short, ridges forming discontinuous dorsolateral fold; skin on flanks shagreen with small scattered tubercles, skin on throat smooth, skin on chest and belly weakly areolate; discoidal fold absent, weak thoracic fold present; cloacal sheath short; large tubercles absent in cloacal region. Outer surface of forearm without minute tubercles; outer palmar tubercle bifid, low, ovoid, about twice the size of ovoid inner palmar tubercle; supernumerary tubercles indistinct in preservative; subarticular tubercles low, ovoid, most prominent on base of fingers; fingers without lateral fringes; Finger I shorter than Finger II; tips of digits rounded, lacking marginal grooves (Fig. 7C).

Hind limbs long and slim, TL 49.3% of SVL; FL 49.8% of SVL; upper surface of hind limbs tuberculate with narrow diagonal ridges (Fig. 10A); posterior and ventral surfaces of thighs areolate; heel with a distinct conical tubercle; outer surface of tarsus with small tubercles; outer metatarsal tubercle round, about twice as large as ovoid inner metatarsal tubercle; supernumerary plantar tubercles indistinct in preservative; subarticular tubercles low, ovoid in dorsal view, most distinct on base of toes; toes without lateral fringes; basal webbing absent; toe tips rounded, lacking marginal grooves, about as large as those on fingers; relative lengths of toes: 1 < 2 < 3 > 5 < 4; Toe V shorter than Toe III (Fig. 7D).

Measurements (in mm) of holotype:SVL 20.7; TL 10.2; FL 10.3; HL 6.9; HW 7.0; ED 2.4; IOD 2.3; EW 1.9; IND 2.1; E-N 1.8.

#### Coloration of holotype in life.

(Fig. 8): Dorsum reddish brown with dark gray and yellowish-brown mottling, and few white tubercles; dark brown interorbital bar bordered anteriorly with a narrow yellowish-brown stripe; dorsal surface of forearms and hind legs with diagonal dark brown bars outlined with pale grayish-brown stripes; dark brown canthal stripe; broad, dark brown supratympanic stripe outlined by tan; broad, dark brown bar on upper lip below eye, bordered by narrow, tan stripe on each side; flanks colored as dorsum with a broad gray diagonal stripe at the anterior half, bordered on both sides with a narrow tan stripe; axilla and groin brown and gray mottled; gray inguinal bar bordered on both sides with a narrow tan stripe extending onto dorsal surface of thigh; posterior surfaces of thighs and concealed surfaces of shanks brown and gray mottled; throat gray with pale gray mottling; chest, belly, and extremities gray with pale gray dots and flecks; outer fingers and outer toes yellowish brown, palmar and plantar surfaces dark gray; iris predominately reddish brown with fine black reticulations and golden ringlet around pupil.

#### Coloration of holotype in preservative.

(Figs 5C, D): As described above but slightly paler with gray coloration being brown and yellowish brown being white; iris gray.

#### Variation.

Freshly hatched froglets (FMNH 282820-22) had a SVL of 6.2–6.5 mm (n = 3). Froglets were pale green with brown blotches on dorsum, brown canthal and supratympanic stripes, brown bars on upper lip, and distinct diagonal brown stripe on flanks and on extremities (Fig. 10C), all brown bars and stripes bordered with white stripes.

#### Etymology.

The specific name *curator* is the Latin noun meaning caretaker and refers to the observed behaviour of the female guarding nine terrestrial eggs.

#### Distribution, natural history, and threat status.

**Figure 9.**
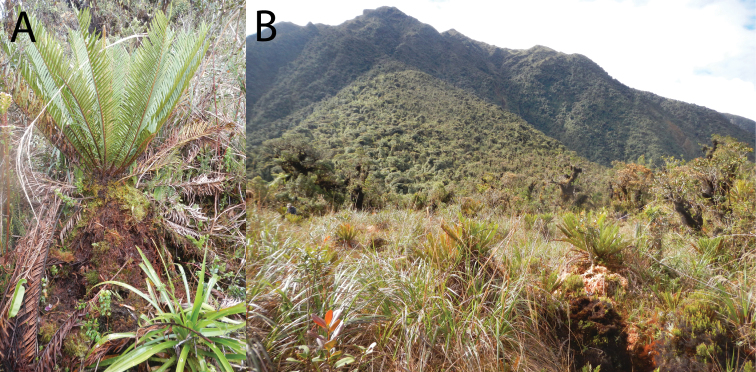
Habitat of *Phrynopus curator*. Fern where *Phrynopus curator* and clutch were found (**A**) on pampa of Quebrada Yanachaga at 3000 m elevation (**B**). Photos by E. Lehr.

**Figure 10.**
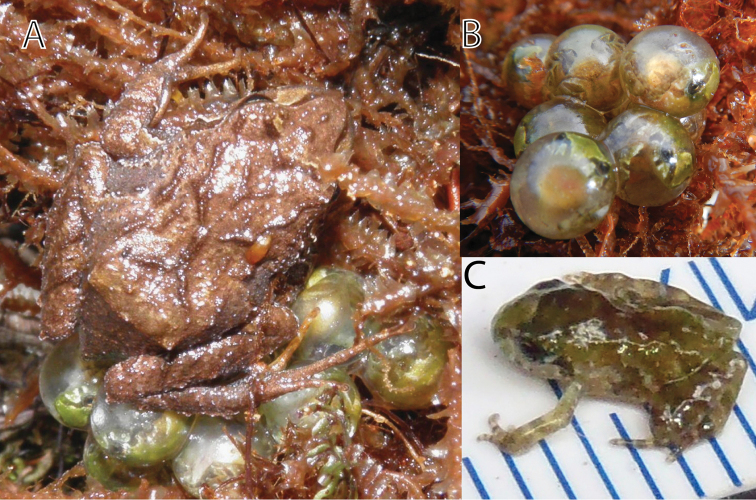
*Phrynopus curator* (MUSM 31106) guarding eggs (**A**), eggs (**B**) and hatchling (FMNH 282820-22) with scale in mm (**C**). Photos by J. Moravec (**A, C**) and E. Lehr (**B**).

The species is only known from the type locality (a summit of a mountain ridge of Cordillera Yanachaga, 3000 m). The habitat consisted of transitional formation between cloud forest and wet puna. The type locality is a slightly swampy plain covered by scattered bushes, small isolated trees, ferns, moss, and lichens (Fig. 9). The single specimen was found at 3.30 pm under a moss layer at approx. 10–15 cm depth covering the stem of a fern. The female was gravid (small unpigmented eggs) and guarding a clutch consisting of nine terrestrial eggs and was partially sitting on them (Fig. 10A). Young frogs (Fig. 10B) were clearly visible based on their pale green coloration and moved inside the eggs when being disturbed. The eggs had a diameter of 5.7–5.8 mm (n = 3). Two days after collection of three eggs three froglets hatched. *Phrynopus curator* called from under moss during the day. Sympatric anurans include *Gastrotheca* sp. (heard call), *Pristimantis aniptopalmatus*, *Pristimantis bromeliaceus* and *Pristimantis* sp. We classify *Phrynopus curator* as “Data Deficient” according to the IUCN red list criteria and categories based on the limited information on its geographic range.

## Discussion

Despite the limited number of specimens of *Phrynopus badius* and *Phrynopus curator* both species are morphologically clearly differentiated from all other known species of *Phrynopus* which justifies their description. Calling males of both species were heard by day in leaf litter and moss layer. However, despite our efforts males were not located in the dense vegetation. Both species of *Phrynopus* occupy different habitats. Whereas *Phrynopus badius* was found on the steep mountain slopes under closed canopy of cloud forest, *Phrynopus curator* inhabited moss layers in open transitional area between cloud forest and wet puna. Both species seem to display a dissimilar adaptation to different habitat conditions. *Phrynopus badius* has shorter legs (holotype TL 37.2% of SVL, paratype TL 35.7% of SVL) and moves by walking or short jumps. On the contrary, *Phrynopus curator* has longer legs (TL 49.3% of SVL), fingers and toes, is more agile and jumps readily if disturbed.

With *Phrynopus curator* we describe and document the first clutch and case of parental care for a species of *Phrynopus*. A previous report of parental care was provided by Catenazzi (2006) for a former member of the genus *Phrynopus* (*Phrynopus cophites*, now placed in the genus *Bryophryne)*. Clutches of strabomantid frogs and strabomantid frogs performing parental care are rarely found. In some species parental care is conducted by females only, while in others it is restricted to males and in a few both sexes show parental care (see Duellman and Lehr 2009). In the case of *Phrynopus curator* we found that females take care for eggs until they complete development. The observed female was sitting on the clutch motionless, with its belly and toes having direct contact with egg surfaces. The fact that the female was gravid (small ovarian eggs) indicates a fast reproductive cycle of this species. Because the stomach of the female was completely filled with unidentified parts of arthropods, food must have been consumed during the guarding period. However, it remains unknown whether the female leaves the clutch for this purpose or feeds on nearby arthropods.

New species of *Phrynopus* are frequently discovered when field work is conducted in cloud forests and puna regions in central Peru (e.g. Lehr and Oróz 2012). Therefore, the species diversity in this genus is highly underestimated. The currently known 25 species of *Phrynopus* are allocated to the departments of La Libertad (1 species), Huánuco (10), Pasco (11), and Junín (4) (Lehr and Oroz 2012, this paper). With seven known species (Fig. 3), the YCNP has the highest regional species diversity of *Phrynopus*, and we expect further new species if herpetological surveys are continued in the YCNP.

## Supplementary Material

XML Treatment for Phrynopus badius

XML Treatment for Phrynopus curator

## Appendix I

**Specimens examined**

*Phrynopus bracki*: PERU: Pasco: Pasco: Parque de la Nación Yanachaga-Chemillén, 2600 m: MUSM 4400 (paratype); Parque de la Nación Yanachaga-Chemillén: San Alberto: MUSM 19906–08.

*Phrynopus dagmarae*: PERU: Huánuco: Palma Pampa, 3020 m: MUSM 20451 (holotype).

*Phrynopus horstpauli*: PERU: Huánuco: Ichocan, Jatunloma-forest, 3100 m: MTD 44333–39.

*Phrynopus interstinctus*: PERU: Húánuco: Cordillera de Carpish, San Marcos, 3100 m: MUSM 29543 (holotype), 3160 m: MUSM 29544–29545 (paratypes).

*Phrynopus peruanus*: PERU: Junín: Puna of Maraynioc (11°21'35.2"S, 75°28'52.6"W), 3825 m: MUSM 19977–78.

*Phrynopus vestigiatus*: PERU: Húánuco: Cordillera de Carpish, San Pedro de Carpish, 3100 m: MUSM 29542 (holotype).
